# The efficacy and safety of stepwise oral food challenge in children with hen’s egg allergy

**DOI:** 10.1186/s13223-024-00941-4

**Published:** 2024-12-18

**Authors:** Mika Ogata, Jun Kido, Takanobu Yoshida, Natsuko Nishi, Sachiko Shimomura, Nami Hirai, Tomoyuki Mizukami, Masaaki Yanai, Kimitoshi Nakamura

**Affiliations:** 1 Department of Pediatrics, Graduate School of Medical Sciences, Kumamoto University, Kumamoto City, Japan; 2 Department of Pediatrics, National Hospital Organization Kumamoto Medical Center, Kumamoto City, Japan; 3 Department of Pediatrics, Kumamoto University Hospital, 1-1-1 Honjo, Kumamoto City, 860-8556 Japan; 4 Department of Pediatrics, Kumamoto Regional Medical Center, Kumamoto City, Japan; 5 Kumamoto Pediatric Allergy and Immunology Study Group, Kumamoto City, Japan

**Keywords:** Hen’s egg allergy, Oral food challenge, Oral immune tolerance, Stepwise oral food challenge

## Abstract

**Background:**

Oral food challenge (OFC) is the gold standard for diagnosing food allergies (FAs) but carries the risk of anaphylactic reaction. Stepwise OFC, starting with a low dose of allergen and progressing to medium and full doses, is effective in determining a tolerable dose. We retrospectively evaluated the results of a stepwise OFC for hen’s egg (HE) to demonstrate its safety and efficacy. We discuss whether early low-dose administration of HE induces early immune tolerance in HE allergy.

**Methods:**

We included 2,058 children (median, 2.6 years) who underwent HE-OFC between 2017 and 2021 at two institutes in Japan. The target challenge dose of OFC was classified as low (less than 1/8 of a cooked egg), medium (1/8 or more but less than 1/2), or full (1/2 or more). If the low-dose OFC was negative, subjects were allowed to consume the same dose of HE and underwent medium-dose OFC within 12 months. Even if positive, individuals were recommended to consume previously-tolerated amounts of HE and repeat OFC at the same dose within 12 months. We evaluated the correlation between their OFC results and response.

**Results:**

A total of 526 (25.6%) children presented positive reactions. There were no cases of anaphylactic shock. Higher serum egg white (EW)- (P < 0.001) and ovomucoid (OVM)- specific IgE (P < 0.001) (sIgE) levels were associated with positive OFC. The low-dose OFC group had more positive reactions (*P* < 0.001), younger children (*P* < 0.001), higher EW-sIgE (*P* < 0.001) and OVM-sIgE (*P* < 0.001), and more histories of anaphylaxis (*P* = 0.014). OFC-positive children were younger than OFC-negative children, particularly in low-dose OFC (*P* = 0.010). OFC results between complete and partial elimination of HE groups across all EW- or OVM-sIgE classes were similar (*P* > 0.05).

**Conclusions:**

Stepwise OFC is safe and effective in diagnosing HE allergy and facilitates the earlier introduction of HE in children. This study suggests the limited potential of early consumption of lower doses of HE to induce earlier immune tolerance, such that other strategies to induce earlier tolerance in infants with HE allergy should be considered.

**Supplementary Information:**

The online version contains supplementary material available at 10.1186/s13223-024-00941-4.

## Background

Hen’s egg (HE) is one of the most common causative agents behind IgE-mediated food allergy (FA) in children [1–3]. HE is commonly used in various types of cooked food. Thus, it is difficult for children with HE allergies to completely avoid HE, leading to economical and psychological burdens on them and their families [4, 5]. Children can often outgrow HE allergy by school age [6], and many children with HE allergy can tolerate boiled or baked HE [7, 8]. If children with HE allergy can tolerate even a small amount of HE, it can improve their quality of life (QOL) and that of their parents [9]. Therefore, it is crucial for healthcare professionals to diagnose HE allergy correctly in children and identify the tolerated doses that can be safely ingested.

Oral food challenge (OFC) is the gold standard for diagnosing FA [10]. However, OFC involves the risk of anaphylactic reactions and stress for performers of the OFC test. This is why its implementation by general pediatricians is limited [11–13]. Although serum specific IgE (sIgE) or skin prick tests may identify IgE sensitization for suspicious food allergens, their interpretation is generally difficult without definite histories of allergic reactions [14]. A 95% positive predictive value (PPV) is the cut-off level for sIgE indicating 95% positivity in an OFC-positive result, whereas a 50% negative predictive value (NPV) indicates the cut-off level for sIgE showing 50% negativity in OFC-negative results. Several PPVs/NPVs have been reported as alternative diagnostic parameters for OFC [14, 15]. However, many cases showed lower PPVs and higher NPVs. Moreover, recommended cutoff values may vary in each study due to differences in the patient population and disease prevalence [14, 15]. Basophil Activation Testing (BAT) is also available to diagnose HE allergy [16]. However, it cannot indicate the threshold dose. Therefore, OFC is usually required for definitive diagnosis [14, 17].

In Japan, stepwise OFC [18], which is considered safe and can use definable doses [19], has been recommended such that OFC is performed in specialist allergy units as well as in general hospitals and clinics throughout the nation [3, 20].

In the present study, we retrospectively evaluated the results of a stepwise OFC for HE performed in children to demonstrate the safety and efficacy of using stepwise OFC for diagnosing HE allergy. Additionally, we discuss whether early low-dose administration of HE can contribute to inducing early immune tolerance or desensitization in children with HE allergy.

## Methods

### Study Population

We selected 2,058 children (median age, 2.6 y; interquartile range, 1.6–4.8 y) among 6,929 children who underwent OFC between January 1, 2017, and December 31, 2021, at the Department of Pediatrics of the National Hospital Organization Kumamoto Medical Center and Kumamoto Regional Medical Center, both located in Kumamoto Prefecture, western Japan. The children were checked for their serum total IgE, egg white (EW)-sIgE, and ovomucoid (OVM)-sIgE levels within 1 year of undergoing their first OFC (Supplementary Data 1).

### Data Collection

We retrospectively extracted laboratory data, OFC test results, age at OFC, and medical history from hospital records. Serum EW-sIgE and OVM-sIgE levels were measured using ImmunoCAP systems (Thermo Fisher Diagnostics, Uppsala, Sweden). *Classes of sIgE are shown in Supplementary Data* 2. Medical history included a history of immediate symptoms and anaphylaxis to HE, atopic dermatitis (AD) requiring treatment, and a history of bronchial asthma (BA), including wheezing. BA history was characterized by occurrences of wheezing episodes in the past and the need for beta-2 agonist inhalation therapy at least once; wheezing due to OFC or accidental ingestion of HE was not considered. Some participants underwent OFC more than once during the study period due to increased dosing or the need for repeat testing in case of unclear OFC results, such as when minor, subjective symptoms appeared or the results could not be confirmed. In such cases, the OFC performed on the same individual on a different day was considered a separate OFC event in this study. Anaphylaxis was diagnosed and treated according to the World Allergy Organization (WAO) guidelines [21].

### OFC tests

The OFC test was conducted in an open, unblinded design according to the 2017 and 2020 Japanese guidelines for FA [3, 22]. The definition of the positive OFC was based on these guidelines. If the children developed immediate reactions after ingestion of the causative food in the OFC test, we considered this condition as OFC-positive and discontinued subsequent dosing. Children without allergic symptoms in the OFC test were considered negative. The severity of the OFC reaction was assessed based on the most severe manifestations during the OFC test using the WAO anaphylaxis grading scale [21].

The target challenge doses of OFC were classified as low (less than 1/8 of a cooked HE or less than 0.45 g of HE protein), medium (1/8 or more but less than 1/2 of a cooked HE), or full (1/2 or more of a cooked HE, or 1.8 g or more of HE protein) [23]. An HE yolk challenge test was performed if participants had recently reacted to a medium or high dose of HE or had class 5 (≥ 50 kU/L) levels of EW- or OVM-sIgE. A cooked HE was a 20-min boiled EW (containing 3.6 g of HE protein) prepared by their caregivers. We administered the target challenge doses as multiple fragmented doses every 30–60 min. Since some younger children had difficulty in ingesting HE or children with high anxiety refused to consume an amount of HE, we had to discontinue some OFC tests after a single dose. As a general rule, the participants first underwent a low-dose OFC. If they passed this OFC, the subjects were allowed to consume HE up to the tolerated dose 2–3 times per week; the next dose of OFC with an increased total challenge dose was conducted within 12 months [3, 22, 24].

Even with a positive OFC result, individuals were recommended to continue consuming previously-tolerated amounts of HE, including processed eggs [3] and underwent a repeat OFC with the same total challenge dose within 12 months [18].

### Statistical analysis

**Fig. 1:**
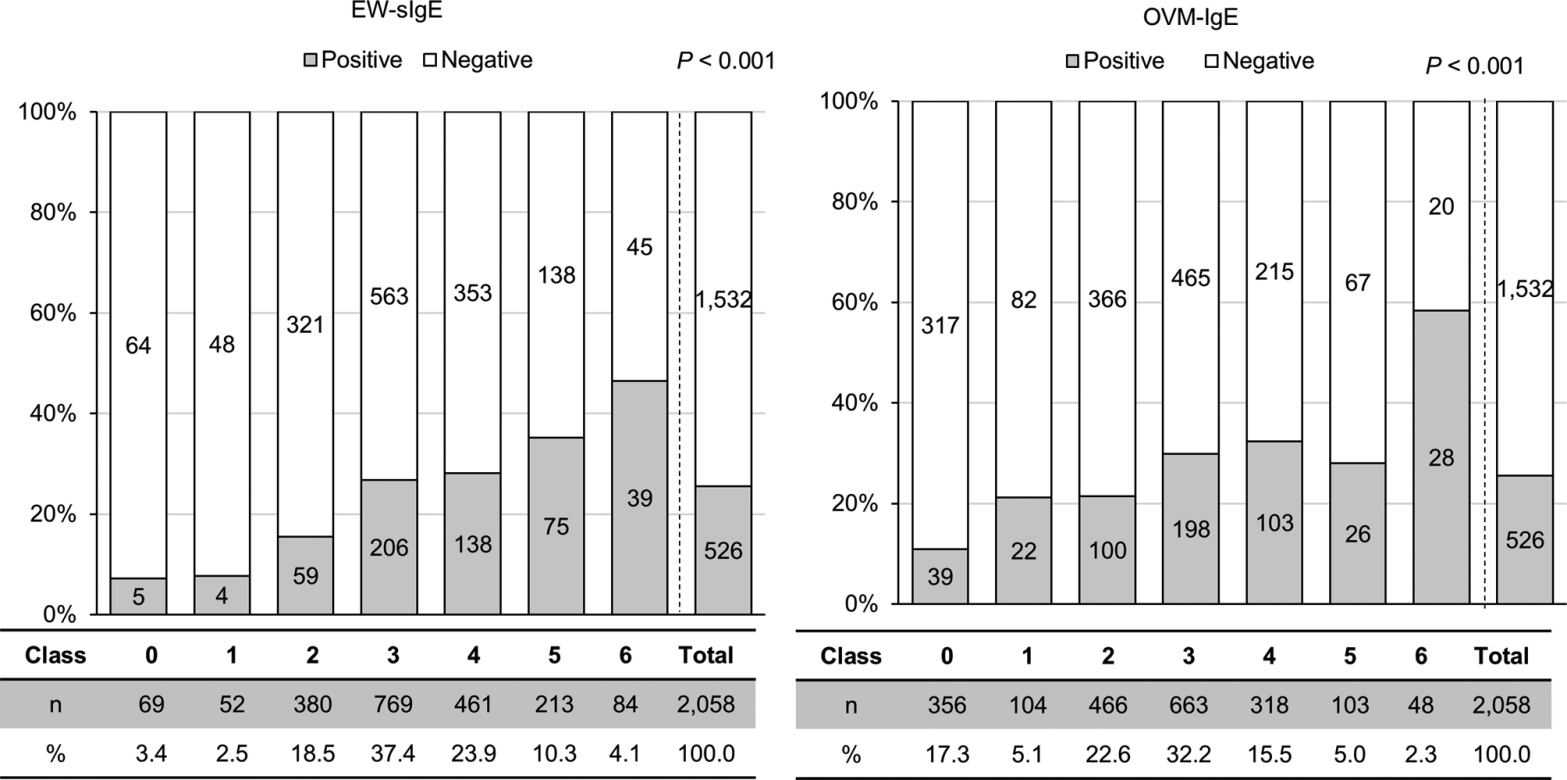
EW- or OVM-sIgE levels and OFC results. HE, hen’s egg; OFC, oral food challenge test; sIgE, serum-specific IgE; EW, egg white; OVM, ovomucoid

**Fig. 2:**
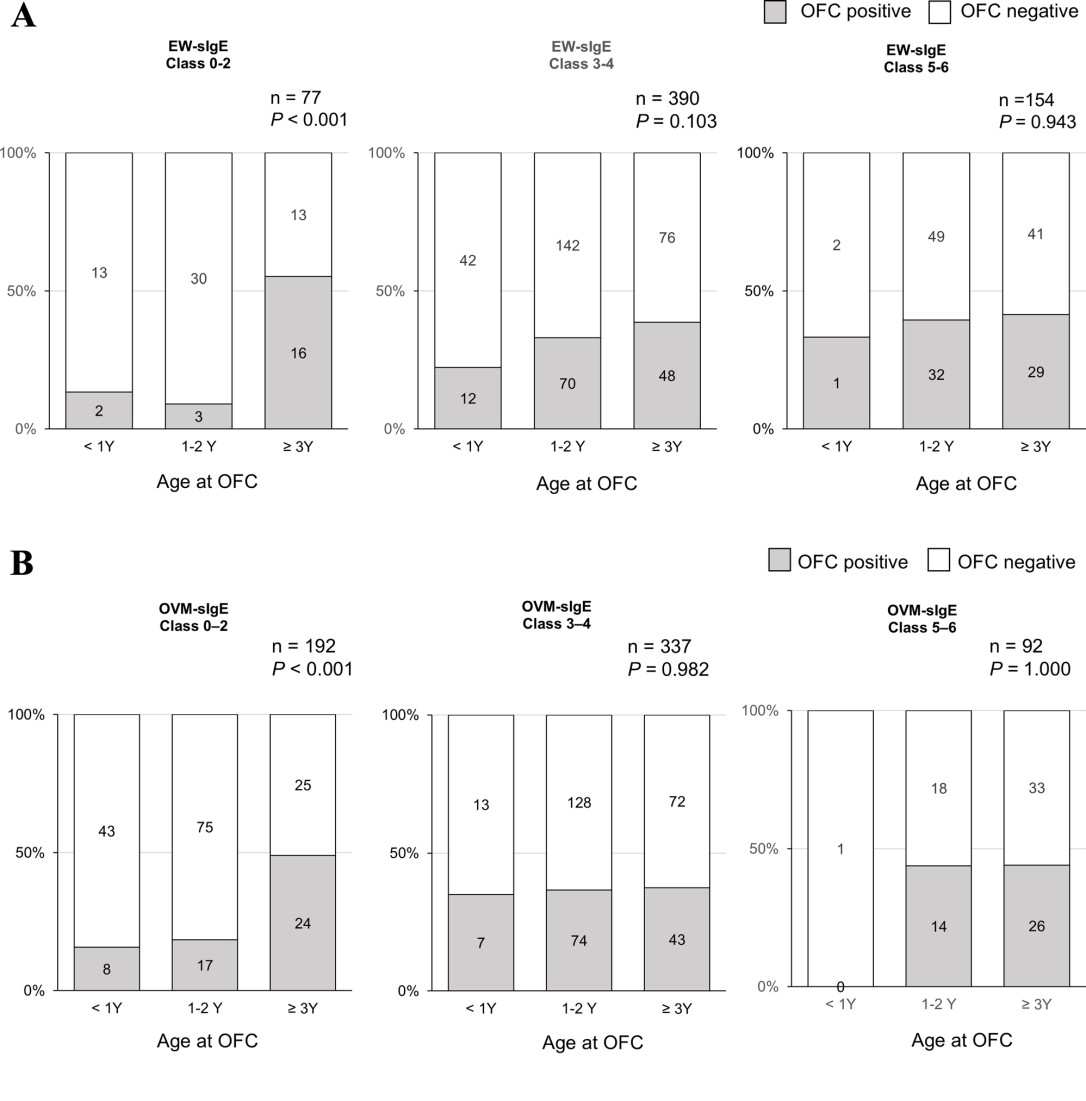
Results of the low-dose OFCs categorized by each EW- or OVM-sIgE class and age group. (**A**) EW-sIgE, (**B**) OVM-sIgE. HE, hen’s egg; OFC, oral food challenge test; sIgE, serum-specific IgE; EW, egg white; OVM, ovomucoid

**Fig. 3:**
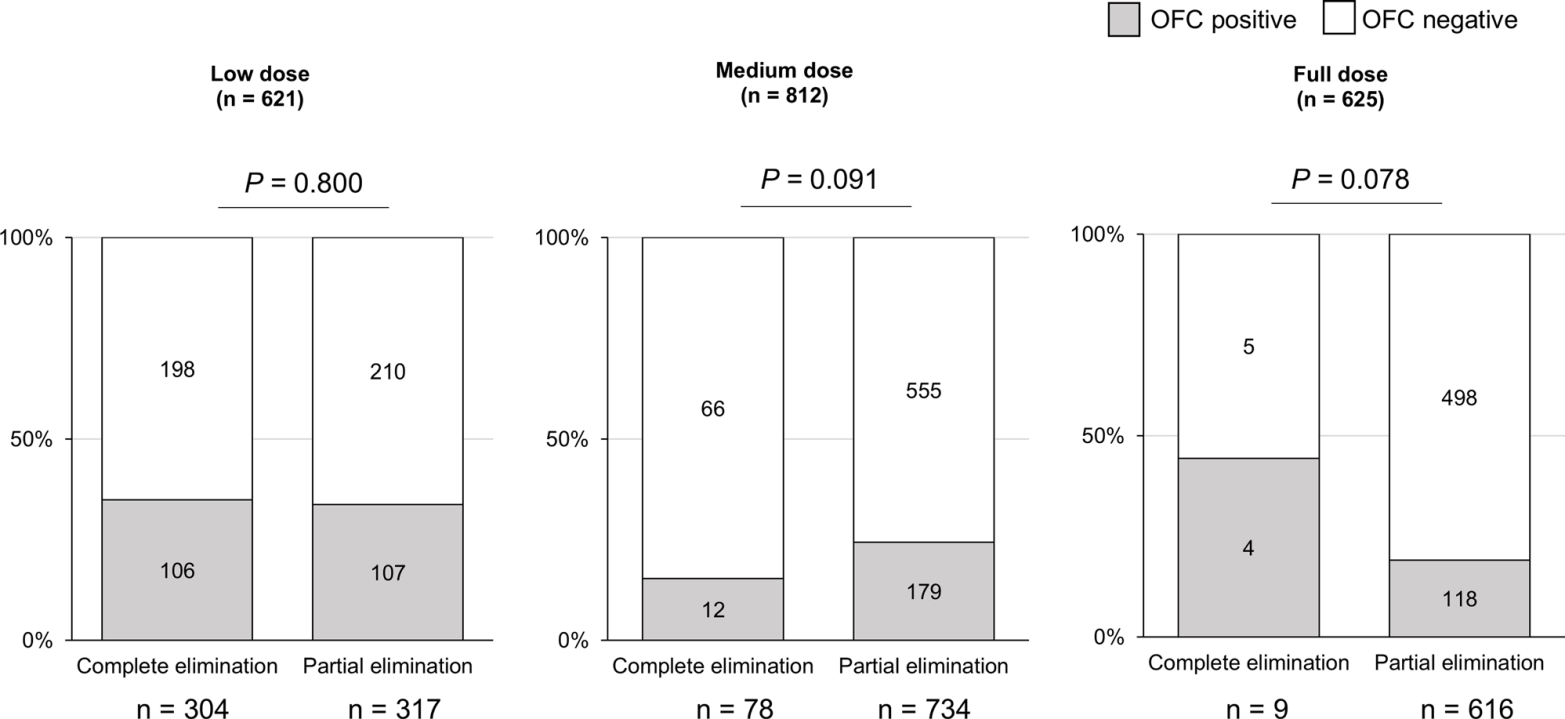
OFC results in participants with complete or partial elimination of HEs. HE, hen’s egg; OFC, oral food challenge test; sIgE, serum-specific IgE; EW, egg white; OVM, ovomucoid

**Fig. 4:**
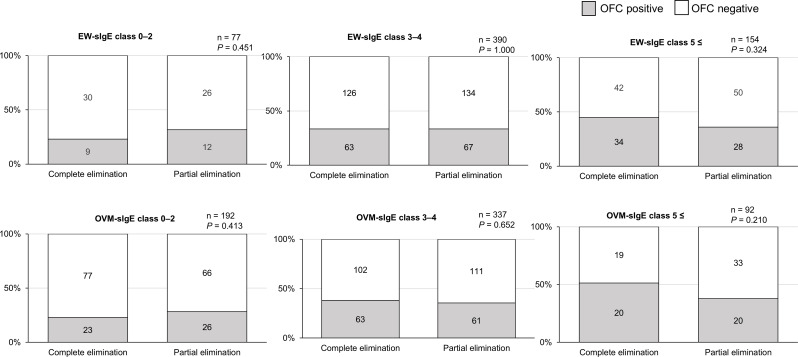
Results of the low-dose OFCs for each EW- and OVM-sIgE class (complete vs. partial elimination). HE, hen’s egg; OFC, oral food challenge test; sIgE, serum-specific IgE; EW, egg white; OVM, ovomucoid

The correlations between each ordinal variable (age or HE-specific IgE levels or target challenge doses) and the OFC positivity rates were evaluated using the Cochran–Armitage test (Figs. 1 and 2; Supplementary Data 3, 7, 8) The categorical variables, such as HE-OFC outcomes, immediate reaction history, anaphylaxis history, BA history, active AD, and complete elimination of HE prior to the OFC, were compared between the groups using Fisher’s exact test (Tables 1 and 2; Figs. 3 and 4, Supplementary Data 4, 5; 10) and are presented as numbers and frequencies. The continuous variables, including ages of individuals, total IgE levels, and EW- and OVM-sIgE levels, were compared between three groups using the Kruskal–Wallis test (Tables 1 and 2) or between two groups using the Mann–Whitney U-test (Supplementary Data 5) and are presented as medians and interquartile ranges. Statistical significance was set at *P* < 0.05. All statistical analyses were conducted using EZR (Saitama Medical Center, Jichi Medical University, Saitama, Japan), which is a graphical user interface in R (The R Foundation for Statistical Computing, Vienna, Austria).

**Table 1:** Characteristics for each challenge dose group

Total challenge dose	Low dose	Medium dose	Full dose	Total	***P***-value
N (%, total)	621 (30.2%)	812 (39.4%)	625 (30.4%)	2058 (100.0%)	-
OFC positive (%)	213 (34.3)	191 (23.5)	122 (19.5)	526 (25.6)	< 0.001
Anaphylactic reaction (%)	47 (7.6)	41 (5.0)	24 (3.8)	112 (5.4)	0.014
Age in months [IQR]	24.0 [15.0–49.0]	30.5 [19.0–58.0]	37.0 [25.0–60.0]	31.0 [19.0–57.0]	< 0.001
History of anaphylaxis to HE (%)	128 (20.6)	158 (19.5)	67 (10.7)	353	< 0.001
History of immediate reaction to HE (%)	454 (73.1)	618 (76.1)	484 (77.4)	1556 (75.6)	0.191
Complete elimination of HE (%)	304 (49.0)	78 (9.6)	9 (1.4)	391 (19.0)	< 0.001
History of wheezing (%)	217 (35.0)	255 (31.4)	203 (32.5)	675 (32.8)	0.350
Atopic dermatitis (%)	451 (72.7)	560 (69.0)	402 (64.3)	1413 (68.7)	0.006
Total IgE (IU/mL) [IQR]	206 [78.3–635]	242 [78.0–901]	183 [65.0–643]	217 [73.0–722]	0.034
EW-sIgE (kU/L) [IQR]	19.6 [7.1–48.6]	11.7 [4.1–29.4}	5.5 [1.9–16.7]	11.0 [3.56–30.1]	< 0.001
OVM-sIgE (kU/L) [IQR]	9.9 [2.5–29.5]	4.8 [1.0–14.3]	2.2 [0.4–6.7]	4.53 [0.88–15.3]	< 0.001

OFC, oral food challenge test; HE, hen’s egg; EW, egg white; OVM, ovomucoid, sIgE, serum-specific IgE; IQR, Interquartile range

**Table 2:** Characteristics for each positive-OFC group

Total challenge dose	Low dose	Medium dose	Full dose	***P***-value
OFC positive (%)	213 (34.3)	191 (23.5)	122 (19.5)	< 0.001
Age in months [IQR]	29.0 [17.0–56.0]	33.0 [22.0–57.0]	41.5 [28.0–65.8]	< 0.001
History of anaphylaxis to HE (%)	52 (24.4)	43 (22.5)	17 (13.9)	0.062
History of immediate reaction to HE (%)	158 (74.2)	145 (75.9)	96 (78.7)	0.664
Complete elimination of HE (%)	106 (49.8)	12 (6.3)	4 (3.3)	< 0.001
History of wheezing (%)	80 (37.7)	58 (30.4)	42 (34.4)	0.295
Atopic dermatitis (%)	149 (70.3)	130 (68.1)	78 (63.9)	0.483
Total IgE (IU/mL) [IQR]	251.0 [99.5–665.0]	356.0 [103.0–924.5]	286.5 [94.0–998.8]	0.318
EW-sIgE (kU/L) [IQR]	21.1 [8.2–57.1]	16.0 [7.0–40.9]	11.8 [3.4–23.7]	< 0.001
OVM-sIgE (kU/L) [IQR]	14.0 [4.1–38.0]	7.4 [2.5–20.9]	4.1 [1.2–11.3]	< 0.001

OFC, oral food challenge test; HE, hen’s egg; EW, egg white; OVM, ovomucoid, sIgE, serum-specific IgE; IQR, Interquartile range

## Results

### Correlation between HE-OFC results and HE-specific IgE levels

Of the 2,058 children with HE allergy, 526 (25.6%) showed positive reactions to HE in the HE-OFC (Fig. 1; Supplementary Data 3). There were no cases of anaphylactic shock. Higher serum EW-sIgE (*P* < 0.001) and OVM-sIgE (*P* < 0.001) levels were associated with positive OFC. Although 7.2% (5/69) of the children with EW-sIgE levels (class 0) presented positive reactions to HE, 2 were untreated, and 3 (multiple hives, 2; itchy mouth and nausea, 1) were successfully treated with antihistamines. Almost half (53.6%, 45/84) of the children with EW-sIgE (class 6) presented no allergic reaction in the OFC. Of these children, 5 could consume the full dose of boiled HE and 1 the medium dose. However, all 6 of these children presented an OVM-sIgE level below class 4 (class 4, 4; class 2, 1; and class 0, 1) and a disparity between EW and OVM-sIgE levels. Even among children included in OVM-sIgE class 6, 40.4% (20/48) were OFC negative. Only 2 children were tolerant to full-dose boiled HE, 4 to medium dose HE, 9 to low dose, and 5 to HE yolk. Supplementary Data 4 shows the characteristics of children with EW- or OVM- sIgE levels ≥ 100 kU/L (class 6).

### Correlation between HE-OFC results and challenge doses

Of the 2,058 children, 621 (30.2%) received the low-dose OFC, 812 (39.5%) received the medium-dose OFC, and 625 (30.4%) received the full-dose OFC (Table 1). Allergic reactions were developed in 34.3% (213/621) of the children receiving the low-dose OFC, 23.5% (191/812) receiving the medium-dose OFC test, and 19.5% (122/625) receiving the full-dose OFC test. The frequency of the positive OFC test was higher in the low-dose OFC test than in the other tests (*P* < 0.001) (Table 1).

The median age of children undergoing the low-dose OFC test was lower than those in the middle-dose OFC and full-dose OFC (*P* < 0.001). Additionally, children in the low-dose OFC group presented higher levels of EW-sIgE (*P* < 0.001) and OVM-sIgE (*P* < 0.001), more histories of anaphylaxis (*P*<0.001), and higher active atopic dermatitis cases (*P* = 0.006) than the other groups. However, the frequency of wheezing history and immediate symptoms were similar (*P* > 0.05).

### Comparison of OFC-positive and -negative subjects in total challenge doses

EW- and OVM-sIgE levels were higher in OFC-positive children than in OFC-negative children in every challenge dose test (Supplementary Data 5). Moreover, the OFC-negative children in the full dose OFC tests presented lower EW-sIgE and OVM-sIgE levels, with a median of 4.8 kU/L (IQR: 1.6–14.3 kU/L) and 1.6 kU/L (0.3–5.5 kU/L), respectively. Children undergoing each OFC test were older in the OFC-positive group than in the negative group; however, statistical significance was confirmed only in the low-dose OFC test (*P* = 0.010).

Of the children with a positive OFC (Table 2), children with a low-dose positive-OFC were younger when the OFC test was performed (*P* < 0.001) and showed higher EW- (*P* < 0.001) and OVM-sIgE levels (*P* < 0.001). Total IgE (*P* = 0.318), the frequency of wheezing history (*P* = 0.295), atopic dermatitis (*P* = 0.483), or immediate symptoms related to HE allergy (*P* = 0.664) were similar among age groups.

Both EW- and OVM-sIgE levels were substantially higher in positive-OFC children than in negative-OFC children in each total challenge dose (Supplementary Data 5). The cut-off values of EW- and OVM-sIgE indicating positive reaction for each OFC dose group were evaluated using ROC curves. The EW- and OVM-sIgE could not clearly predict OFC outcomes, with AUCs less than 0.7 in all OFC dose groups (Supplementary Data 6).

### Can early low-dose OFC safely induce immune tolerance?

We focused on the low-dose OFC group because most children in this study initially underwent low-dose OFC. Although the low dose group included those who could consume low-dose or/and higher (medium- or full-dose) HE, this low-dose group showed more positive reactions and had higher EW- and OVM-sIgE levels than those who underwent the medium- or full-dose OFC test (Table 1). Of the children with class 0–2 EW- and OVM-sIgE levels, older children were more likely to develop allergic reactions to low-dose OFC (*P* < 0.001, Fig. 2A and B).

Even when the children developed positive reactions to low-dose OFC, we encouraged them to ingest a lower dose than the low-dose OFC to avoid the complete elimination of HE. In fact, 51% (317/621) of low-dose challenge cases could tolerate lower doses of HE, such as 1/50–1/200 of a cooked HE. Fewer children completely avoided HE with increasing age (*P* < 0.001, Supplementary Data 7). We hypothesized that intake of a small amount of HE may promote oral immune tolerance to HE in some older children.

We also evaluated the HE-OFC results in children (*n* = 304) who had not consumed HE in any form prior to the OFC and thus had not developed oral immune tolerance. The results were similar by age in each EW- and OVM-sIgE class group (Supplementary Data 8). Moreover, to investigate whether our dietary recommendation of such a trace amount of HE could indeed induce oral immune tolerance in the participants of this study, we compared the OFC results between complete and partial elimination of HE (Fig. 3). In the full dose, a partial elimination was likely to contribute to improving HE allergy, although there was no statistical difference.

In the low-dose OFC group, the ratio of positive OFC results was almost the same between complete and partial elimination groups (*P* = 0.800, 106/304 and 107/317, respectively). We also compared the OFC results between complete and partial elimination groups according to EW- or OVM-sIgE classes (classes 0–2, 3–4, 5–6) (Fig. 4). Although there were no differences (all *P* > 0.05), partial elimination tended to alleviate HE allergy in class 5 or more.

We expected cut-off values of EW- and OVM-sIgE to show positive low-dose OFC results in children who completely eliminated HE from their diet. EW- and OVM-sIgE could not predict OFC results because the receiver operating characteristic (ROC) curve indicated an area under the curve (AUC) < 0.7 (Supplementary Data 9). OFC was required for definite diagnosis in the partial removal group because EW- and OVM-sIgE levels could not be used as indicators. We then compared the results of medium-dose OFC (*n* = 812) in children who went through complete (*n* = 78) and partial (*n* = 734) elimination of HE (Supplementary Data 10). No apparent differences in OFC results between complete and partial elimination groups were observed, and EW- and OVM-sIgE did not contribute to the prediction of OFC results (Supplementary Data 11). These findings were consistent with the results of the low-dose OFC test.

## Discussion

HE is one of the most common causative allergens of FA in children [3]. In this study, we presented the results of a stepwise OFC [24] conducted in children with HE allergy at Kumamoto Medical Center and Kumamoto Regional Medical Center in Kumamoto Prefecture, western Japan.

Stepwise OFC began with the administration of a low dose of HE. A higher percentage of children developed allergic symptoms on the low-dose test than on the medium- or full-dose test. Therefore, the characteristics of the low-dose group can provide valuable guidance when conducting stepwise HE-OFC and may also serve as predictor for stepwise-OFC outcomes. Younger children, those with a history of anaphylaxis, and children with higher levels of EW- or OVM-sIgE were predominant among the low-dose, OFC-positive cases. The EW- or OVM-sIgE levels in children within the high-dose OFC group were lower than those in the low-dose; and the cases with negative high-dose tests had even lower EW- or OVM-sIgE levels. EW- and OVM-IgE levels may provide a prediction of HE-OFC outcomes. However, their sensitivity and specificity are not sufficient for predictive value (Supplementary Data 6). They may be a more useful indicator for being able to undergo high-dose OFC than for receiving low-dose OFC. In low-dose OFC, more children with low EW- or OVM-sIgE level (**<** 3.5 kU/L, class 2) showed positive OFC results with increasing age. Based on the above findings, we are concerned that if children with HE allergy continue to completely avoid HE at age 3 years or later without OFC, tolerance to HE will not be encouraged. In our study population, fewer children aged 3 years or older completely avoided HE compared with children < 3 years (Supplementary Data 7).

Recently, it has been suggested that oral exposure to HE may play a therapeutic role through oral immunotherapy, or a preventive role against HE allergy by early introduction of HE [25] in infancy. Moreover, complete elimination of HE is questionable in children who show mild reaction to a small amount of HE, even when a definite diagnosis is established using an OFC test [26]. Similarly, we believe that even if children cannot tolerate the low-dose HE-OFC test, consumption of lower doses rather than complete elimination of HE may promote their tolerance to HE.

In this study, we evaluated the HE-OFC results only in children (*n* = 304) who had not consumed HE in any form prior to the OFC, and this result did not demonstrate oral immune tolerance among children with partial elimination. There was no difference in the prevalence of positive low-dose OFC by age group among children who avoided HE and had similar OVM-or EW-sIgE levels (Supplementary Data 8), which is different from the results described in Fig. 2. Between complete and partial HE avoidance groups, the OFC positive rates were similar (Figs. 3 and 4). Unfortunately, we could not demonstrate that the consumption of lower doses of HE in children who failed the low-dose HE-OFC would their tolerance to larger amounts. The results of the current study do not demonstrate that younger children who react to low doses of HE are more likely to develop HE tolerance by consuming trace amounts of HE. However, we cannot exclude the possibility that the results may be different if the participants who failed the low-dose OFC consumed a small amount of HE more frequently.

Most children with HE allergy in childhood can outgrow this condition with age [6, 28]. Higher serum sIgE levels may be related to low threshold doses and severe reactions to HE during the OFC [19] and lasting HE allergy [6, 27, 28]. Miyagi et al. suggested that the complete elimination of HE from early infancy for a long time only because of high HE- or OVM-sIgE level may increase the risk of persistent HE allergy, even at school age [29]. Thus, it is important to perform OFC as early as possible to ensure that the child can consume small amounts of HE to reduce anxiety about accidental ingestion. Unfortunately, we could not determine whether early stepwise OFC from infancy would contribute to accelerating tolerance to HE allergy, even in children with very low thresholds who reacted to low doses of HE. More data on efficacy in OFC from early infants should be accumulated.

Other factors apart from HE intake may also induce tolerance. Baked egg products are a safe and effective way to reintroduce HE because of the attenuated antigen. Gallagher [30] reported egg ladders that started with baked egg for infants, including those who had experienced anaphylaxis. We need to explore the strategy using baked egg OFC or food ladders in the future, especially for younger children with HE allergies or suspected HE allergies.

This study had some limitations. We conducted a retrospective study based on the medical records of two institutions in Kumamoto, Japan. The duration between the laboratory blood tests and OFC and the interval between the first and second OFC varied slightly depending on each child due to personal reasons, e.g., a common cold in the child. Laboratory tests other than total IgE and EW-or OVM-sIgE, such as BAT, skin prick test, thymus, and activation-regulated chemokine (TARC), were not evaluated. Moreover, because this was an observational study, the HE-OFC study was performed during a certain period decided by us and we did not describe the clinical course of the participants; we can only recommend suggestions interpreted from the HE-OFC study. Our study design could not demonstrate that stepwise OFC induced immune tolerance. Because immune tolerance means that participants are able to maintain tolerance without regular ingestion, longitudinal data would be required to show this.

## Conclusion

Stepwise HE-OFC is a safe and effective technique for diagnosing HE allergies in children. Introducing low doses of HE during early years may contribute to reducing the anxiety and stress associated with HE allergy in children and their parents. This study suggests the limited possibility of early lower doses of HE consumption inducing earlier immune tolerance. Therefore, other effective strategies to induce earlier immune tolerance for infants with HE-allergy should be considered.

## Electronic supplementary material

Below is the link to the electronic supplementary material.

Supplementary Material 1

## Data Availability

No datasets were generated or analysed during the current study.

## Abbreviations

HE: Hen’s egg
FA: Food allergy
OFC: Oral food challenge test
sIgE: Serum-specific IgE
EW: Egg white
OVM: Ovomucoid
AD: Atopic dermatitis
BA: Bronchial asthma
