# Supplementary material for: The efficacy and safety of stepwise oral food challenge in children with hen’s egg allergy
Source: Allergy Asthma Clin Immunol. 2024 Dec 18;20:67. doi: 10.1186/s13223-024-00941-4 (PMC11658228; doi:10.1186/s13223-024-00941-4)
Supplement: Supplementary file 1 — Supplementary Material 1 [file 13223_2024_941_MOESM1_ESM.docx]

**Supplementary Data**

**Supplementary Data 1.** Study flow chart (upper) and characteristics of participants (lower).

**Supplementary Data 2.**  Classification of sIgE levels (ImmunoCAP®)

**Supplementary Data 3.**  HE- or OVM-sIgE levels and OFC results for each challenge dose group.

**Supplementary Data 4.**  The characteristics of children with EW- or OVM- sIgE levels ≥100 (class 6).

**Supplementary Data 5.**  Differences in characteristics between positive and negative OFC for each challenge dose group

**Supplementary Data 6.** Cut-off levels of HE-sIgE for positive OFC.

The vertical line shows the sensitivity and the horizontal one shows the specificity.

**Supplementary Data 7.** Frequencies of children who had completely avoided eggs prior to low-dose OFCs (n = 621)

**Supplementary Data 8.** Results of low-dose OFCs with complete elimination of eggs by each HE-sIgE class and age group

**Supplementary Data 9.** Cut-off levels of egg-sIgE for low-dose OFC.

The vertical line shows the sensitivity and the horizontal one shows the specificity.

**Supplementary Data 10.** Results of medium-dose OFCs by each EW- and OVM-sIgE class (complete elimination vs. partial desensitization).

**Supplementary Data 11.**  Cut-off levels of HE-sIgE for medium-dose OFC.

The vertical line shows the sensitivity and the horizontal one shows the specificity.

Supplementary Data 1. Study flow chart (upper) and characteristics of participants (lower).

n=2058

Total OFC

2017/1/1–2021/12/31

6,929 cases

Hen’s egg OFC

3,473 cases

**Analyzed**

**2,058 cases**

data unavailability

1,415 cases

| Parameters  median [IQR] | n |
| --- | --- |
| Male (%) | 1302 (63.3) |
| Age (months) | 31.0 [19.0–57.0] |
| Total IgE (IU/mL) | 216.5 [73.0–722.0] |
| EW-sIgE (kU/L) | 11.0 [3.56-30.1] |
| OVM-sIgE (kU/L) | 4.53 [0.88-15.3] |
| History of Immediate reaction to HE(%) | 1556 (75.6) |
| History of Anaphylaxis to HE (%) | 353 (17.2) |
| Complete elimination of HE (%) | 391 (19.0) |
| History of wheezing (%) | 675 (32.8) |
| Atopic dermatitis (%) | 1413 (68.7) |

OFC, oral food challenge; HE, hen’s egg, EW, egg white, OVM, ovomucoid, sIgE,

specific immunoglobulin E

Supplementary Data 2 Classification of sIgE levels (ImmunoCAP®)

| Specific IgE class | Concentration range (kU/L) |
| --- | --- |
| 0 | <0.035 |
| 1 | 0.35 to <0.7 |
| 2 | 0.7 to <3.5 |
| 3 | 3.5 to <17.5 |
| 4 | 17.5 to <50 |
| 5 | 50 to <100 |
| 6 | ≥100 |

sIgE, specific immunoglobulin E

Supplementary Data 3 EW- or OVM-sIgE levels and OFC results for each challenge dose group.

Cochran-Armitage test. The horizontal axis of the graph indicates the class of specific IgE.

EW, egg white; OVM, ovomucoid; sIgE, specific immunoglobulin E

**<OVM-sIgE>**

**<EW-sIgE>**

|  | EW-sIgE ≥100 | OVM-sIgE ≥100 | *p* |
| --- | --- | --- | --- |
| N | 84 | 48 | - |
| Histories of immediate reaction | 61 (72.6 %) | 34 (70.8 %) | 0.843 |
| Histories of anaphylaxis | 14 (16.7 %) | 10 (20.8 %) | 0.640 |
| Complete elimination of HE | 30 (35.7 %) | 17 (35.4 %) | 1.000 |

Supplementary Data 4 The characteristics of children with EW- or OVM- sIgE levels ≥100 (class 6).

Fisher’s exact test.

EW, egg white; OVM, ovomucoid; sIgE, specific immunoglobulin E

| **Total challenge dose** | **The result of OFC** | **N**  **(%)** | **The age of children in months**  **[IQR]** | **History of anaphylaxis to HE (%)** | **History of immediate reaction to HE (%)** | **Complete elimination of HE (%)** | **History of wheezing (%)** | **Atopic dermatitis (%)** | **Total IgE**  **(IU/mL)**  **[IQR]** | **EW-sIgE**  **(kU/L)**  **[IQR]** | **OVM-sIgE**  **(kU/L)**  **[IQR]** |
| --- | --- | --- | --- | --- | --- | --- | --- | --- | --- | --- | --- |
| **Low**  **dose**  **n = 621** | Positive | 213 | 29.0  [17.0 - 56.0] | 52  (24.4) | 158  (74.2) | 106  (49.8) | 80  (37.6) | 149  (70.0) | 251.0  [99.5 – 665.0] | 21.1  [8.2-57.1] | 14.0  [4.1 – 38.0] |
|  | Negative | 408 | 21.5  [15.0 - 56.0] | 76  (18.6) | 296  (72.5) | 198  (48.5) | 137  (33.6) | 302  (74.0) | 194.5  [64.0 – 559.3] | 17.8  [6.1 – 46.5] | 8.5  [1.5 – 26.5] |
|  | *P*-value | - | 0.010 | 0.095 | 0.703 | 0.800 | 0.331 | 0.298 | 0.079 | 0.089 | <0.001 |
| **Middle　dose**  **n = 821** | Positive | 191 | 33.0  [22.0 - 57.0] | 43  (22.5) | 145  (76.2) | 12  (6.3) | 58 (30.4) | 130  (68.1) | 356.0  [103.0 - 924.5] | 16.0  [7.0 - 40.9] | 7.4  [2.5 - 20.9] |
|  | Negative | 621 | 29.0  [18.0 - 59.0] | 115  (18.5) | 473  (75.9) | 66  (10.6) | 197  (31.7) | 430  (69.2) | 217.0  [73.0 - 880.0] | 10.3  [3.1 - 24.6] | 4.1  [0.7 - 12.5] |
|  | *P*-value | - | 0.071 | 0.250 | 0.923 | 0.091 | 0.789 | 0.789 | 0.036 | <0.001 | <0.001 |
| **Full**  **dose**  **n = 625** | Positive | 122 | 41.5  [28.0 – 65.8] | 17  (13.9) | 96  (77.1) | 4  (3.3) | 42  (34.4) | 78  (63.9) | 286.5  [94.0 - 998.8] | 11.8  [3.4 - 11.8] | 4.1  [1.2 – 11.3] |
|  | Negative | 503 | 36.0  [24.0– 60.0] | 50  (9.9) | 388  (78.7) | 5  (1.0) | 161  (32.0) | 324  (64.4) | 167.0  [62.2 - 540.5] | 4.8  [1.6-14.3] | 1.6  [0.3– 5.5] |
|  | *P*-value | - | 0.164 | 0.196 | 0.809 | 0.078 | 0.667 | 0.917 | 0.008 | <0.001 | <0.001 |

Supplementary Data 5. Differences in characteristics between positive and negative OFC for each challenge dose group

The categorical variables, such as history of anaphylaxis, history of immediate reaction, complete elimination of hen's egg (HE) prior to oral food challenge (OFC), history of wheezing, and active atopic dermatitis, were compared between groups using Fisher's exact test and are presented as numbers and frequencies. Continuous variables, including individual age, total IgE levels, and egg white (EW) and ovomucoid (OVM) specific IgE (sIgE) levels, were compared using the Mann-Whitney U test (Table S1) and are presented as medians and interquartile ranges (IQR).

Supplementary Data 6. Cut-off levels of EW- or OVM-sIgE for positive OFC.

| Total challenge dose | N | The cut off level  (kU/L) | | Sensitivity | Specificity | AUC  [IQR] |
| --- | --- | --- | --- | --- | --- | --- |
| Low  dose | 621 | EW-sIgE | 30.300 | 0.418 | 0.652 | 0.542  [0.494-0.589] |
|  |  | OVM-sIgE | 1.880 | 0.869 | 0.265 | 0.591  [0.545-0.637] |
| Medium dose | 812 | EW-sIgE | 4.550 | 0.885 | 0.322 | 0.626  [0.584-0.668] |
|  |  | OVM-sIgE | 3.270 | 0.717 | 0.469 | 0.613  [0.569-0.658] |
| Full  dose | 625 | EW-sIgE | 8.150 | 0.582 | 0.650 | 0.634  [0.580-0.689] |
|  |  | OVM-sIgE | 3.520 | 0.549 | 0.644 | 0.616  [0.561-0.672] |

HE,hens egg; OFC, oral food challenge; AUC, Area Under Curve; EW, egg white; OVM, ovomucoid; sIgE, specific immunoglobulin E

The vertical axis shows the sensitivity and the horizontal one shows the specificity.

**<EW-sIgE>**

**<OVMsIgE>**

**Low dose**

**Medium dose**

**Full dose**


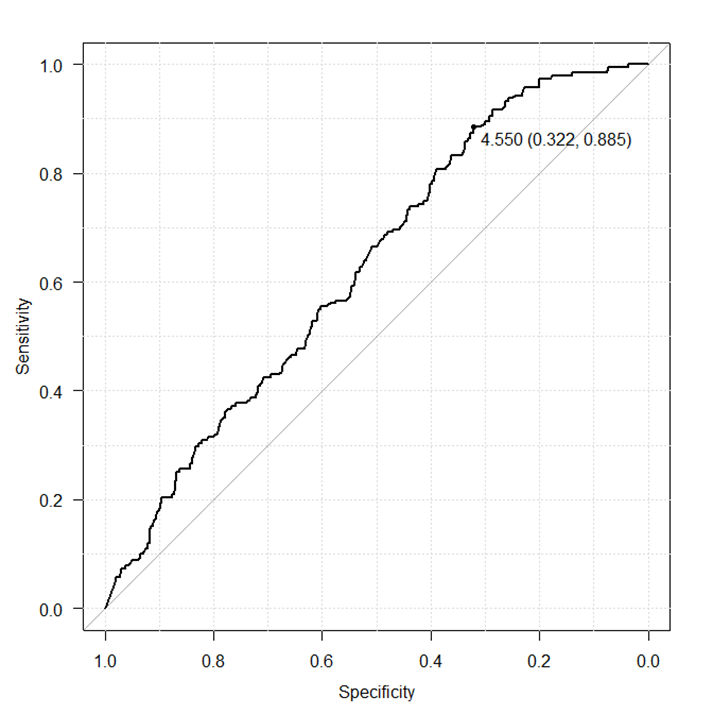

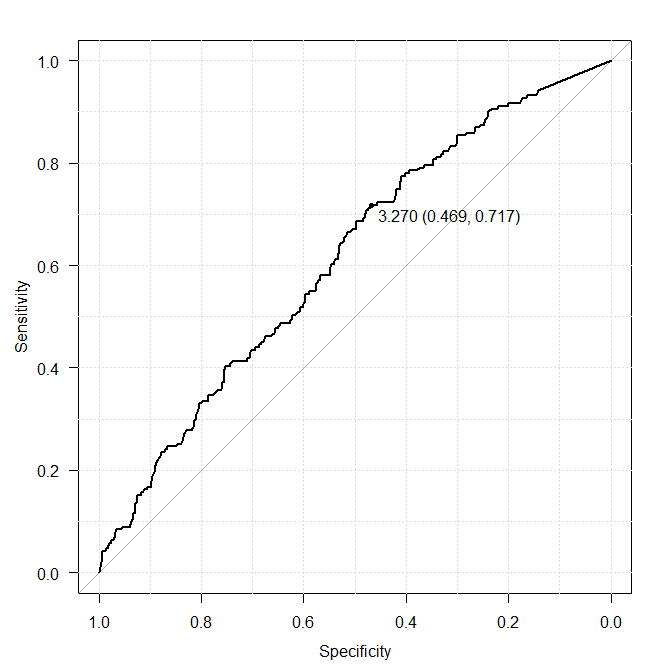

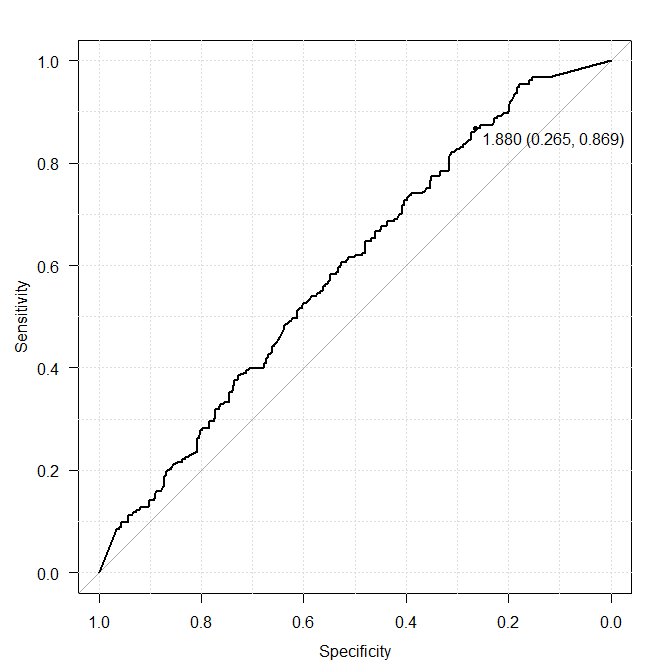

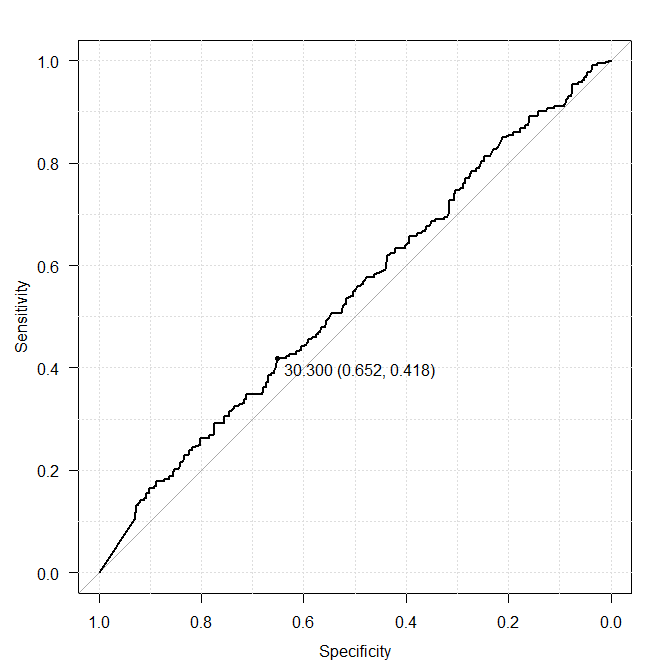

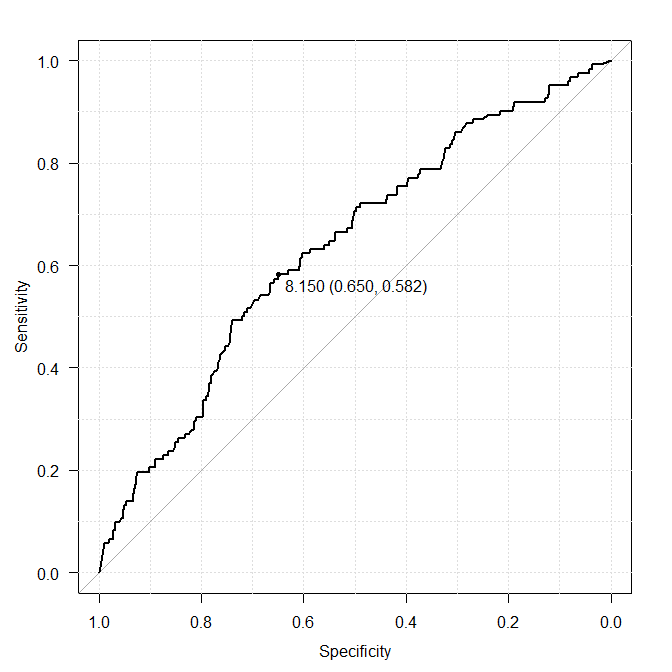

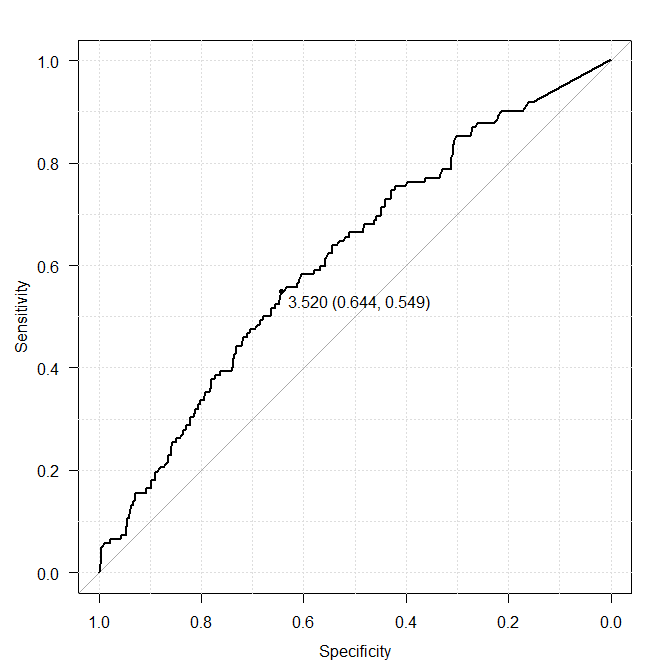


Supplementary Data 7. Frequencies of children who had completely avoided eggs prior to low-dose OFCs.

Age at OFC

Cochran-Armitage test. OFC, Oral food challenge

Supplementary Data 8. Results of low-dose OFCs with complete elimination of eggs by EW-or OVM-sIgE class and age group.

**OVM-sIgE**

**Class 0–2**

**EW-IgE**

**Class 0–2**

**EW-sIgE**

**Class 3–4**

n =189

*P* = 0.191

n = 76

*P* = 0.932

n = 39

*P* = 0.110

n = 39

*P* = 0.634

n = 165

*P* = 0.682

n = 100

*P* = 0.122

**EW-sIgE**

**Class 5–6**

Cochran-Armitage test. OFC, Oral food challenge; EW, egg white; OVM, ovomucoid; sIgE, specific immunoglobulin E

**OVM-sIgE**

**Class 3–4**

**OVM-sIgE**

**Class 5–6**

Supplementary Data 9. Cut-off levels of EW- or OVM-sIgE for low-dose OFC.

**<EW-sIgE>**

**<OVMsIgE>**

**Complete elimination**

**Partial desensitization**


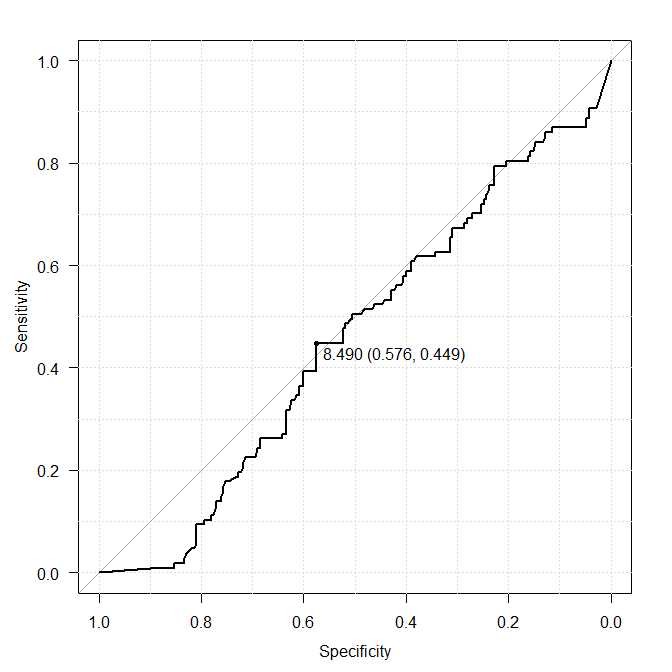

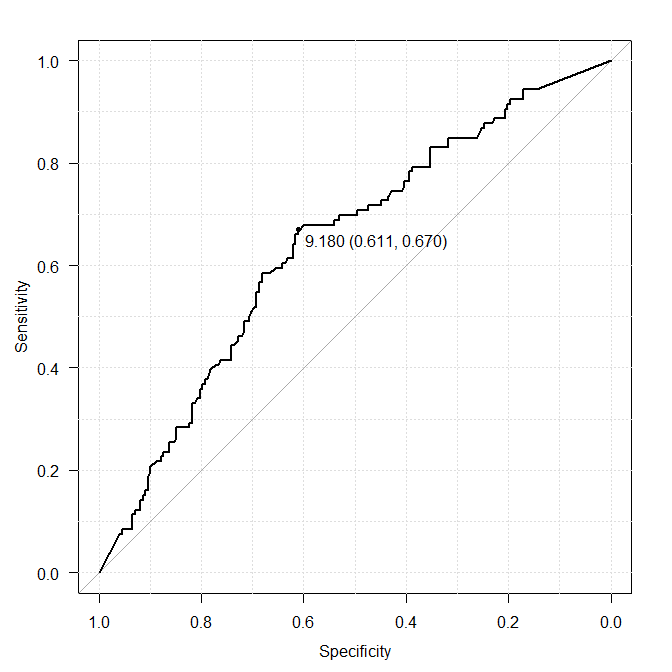

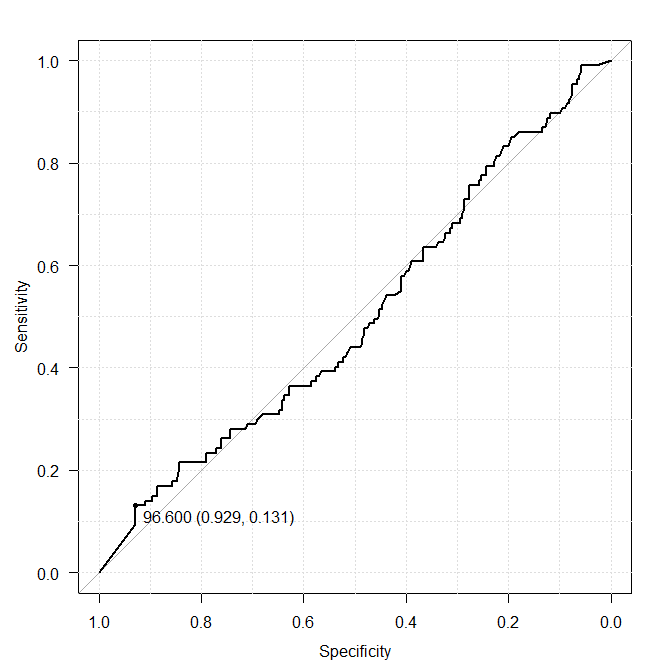

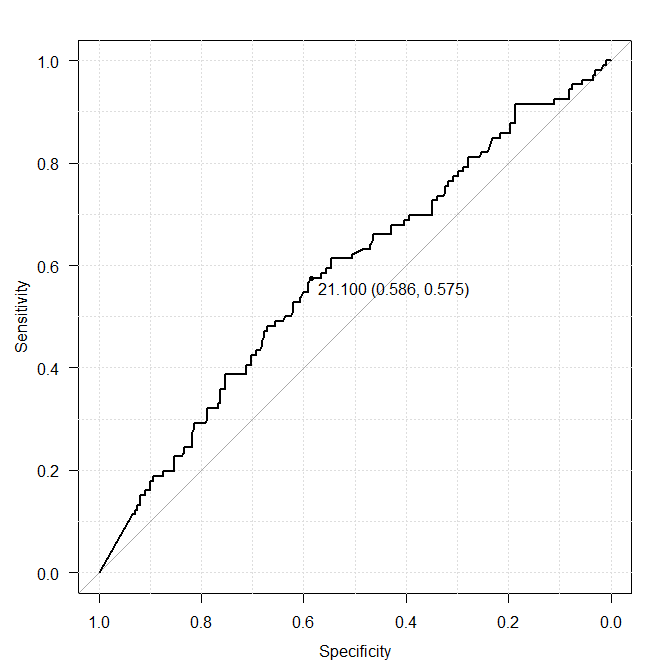

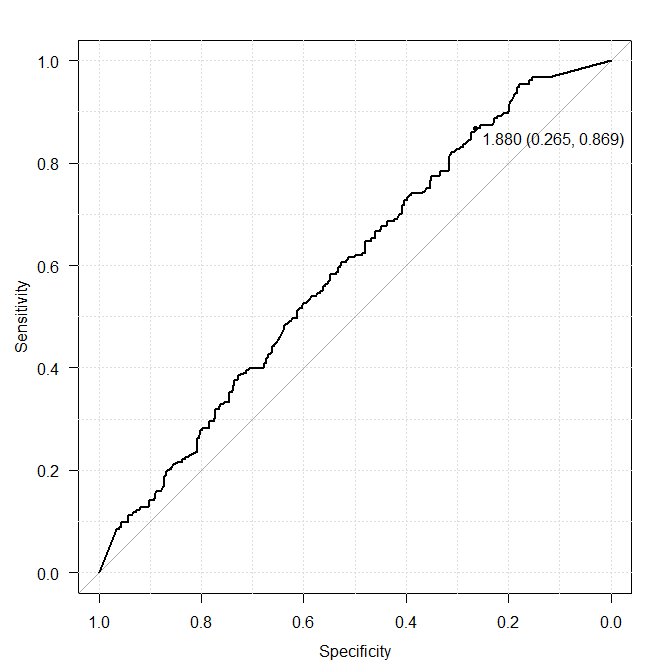

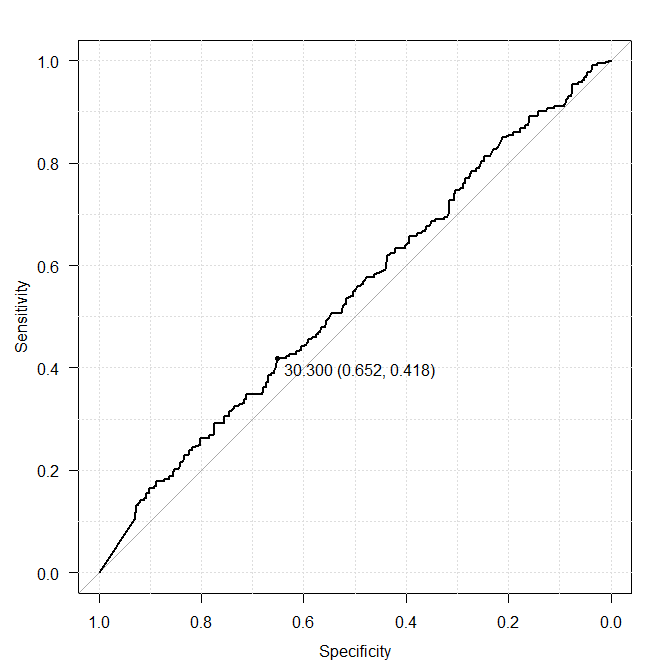


**All**

| Total challenge dose | N | The cut off level  (kU/L) | | Sensitivity | Specificity | AUC  [IQR] |
| --- | --- | --- | --- | --- | --- | --- |
| All | 621 | EW-sIgE | 30.300 | 0.418 | 0.652 | 0.542  [0.494-0.589] |
|  |  | OVM-sIgE | 1.880 | 0.869 | 0.265 | 0.591  [0.545-0.637] |
| Complete elimination | 304 | EW-sIgE | 21.100 | 0.575 | 0.586 | 0.582  [0.514-0.650] |
|  |  | OVM-sIgE | 9.180 | 0.670 | 0.611 | 0.640  [0.575-0.705] |
| Partial elimination | 317 | EW-sIgE | 96.600 | 0.131 | 0.929 | 0.500  [0.432-0.567] |
|  |  | OVM-sIgE | 8.490 | 0.449 | 0.576 | 0.454  [0.389-0.520] |

The vertical axis shows the sensitivity and the horizontal one shows the specificity.

OFC, Oral food challenge; AUC, Area Under Curve; EW, egg white; OVM, ovomucoid; sIgE, specific immunoglobulin E

Supplementary Data 10. Results of medium-dose OFCs by each EW- and OVM-sIgE class (complete elimination vs. partial desensitization).

Fisher’s exact test.

OFC, Oral food challenge; EW, egg white; OVM, ovomucoid; sIgE, specific immunoglobulin E

Supplementary Data 11. Cut-off levels of HE-sIgE for medium-dose OFC.

| Total challenge dose | N | The cut off level  (kU/L) | | Sensitivity | Specificity | AUC  [IQR] |
| --- | --- | --- | --- | --- | --- | --- |
| All | 812 | EW-sIgE | 4.550 | 0.885 | 0.322 | 0.626  [0.584-0.668] |
|  |  | OVM-sIgE | 3.270 | 0.717 | 0.469 | 0.613  [0.569-0.658] |
| Complete elimination | 78 | EW-sIgE | 2.610 | 0.917 | 0.288 | 0.600  [0.424-0.777] |
|  |  | OVM-sIgE | 1.160 | 0.833 | 0.561 | 0.691  [0.517-0.864] |
| Partial elimination | 734 | EW-sIgE | 4.550 | 0.899 | 0.312 | 0.500  [0.432-0.567] |
|  |  | OVM-sIgE | 3.270 | 0.726 | 0.449 | 0.624  [0.580-0.668] |

OFC, Oral food challenge; EW, egg white; OVM, ovomucoid; sIgE, specific immunoglobulin E


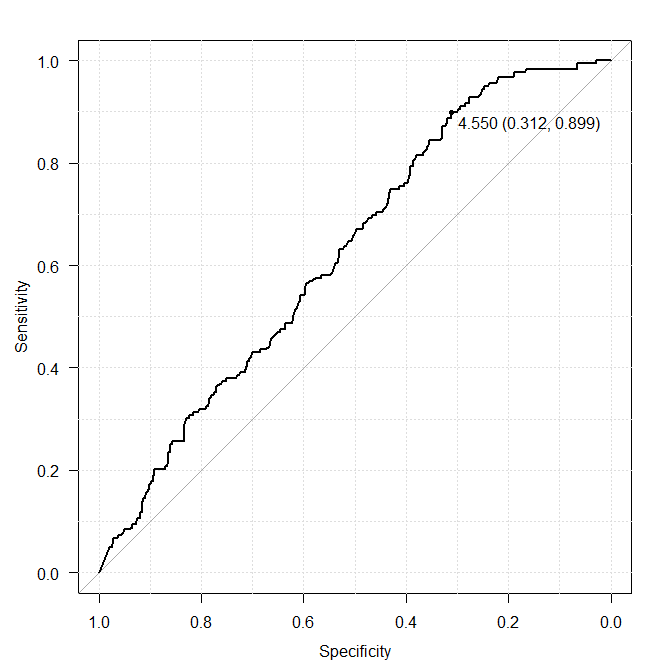

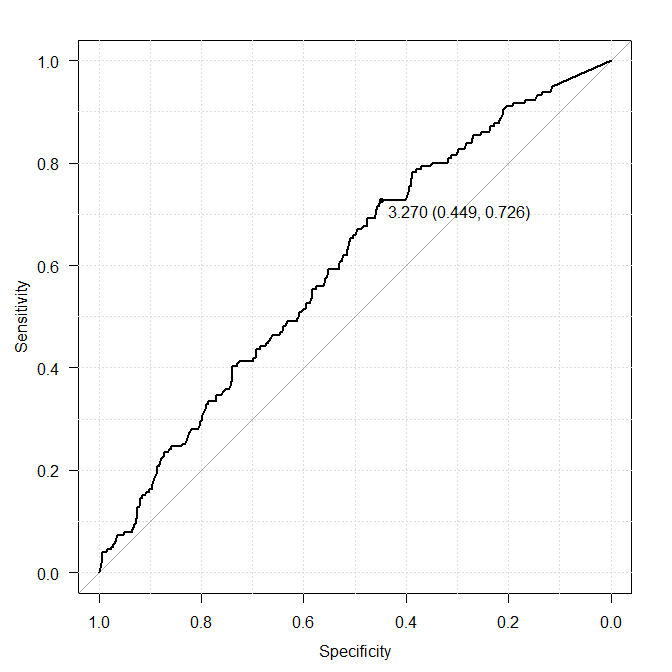

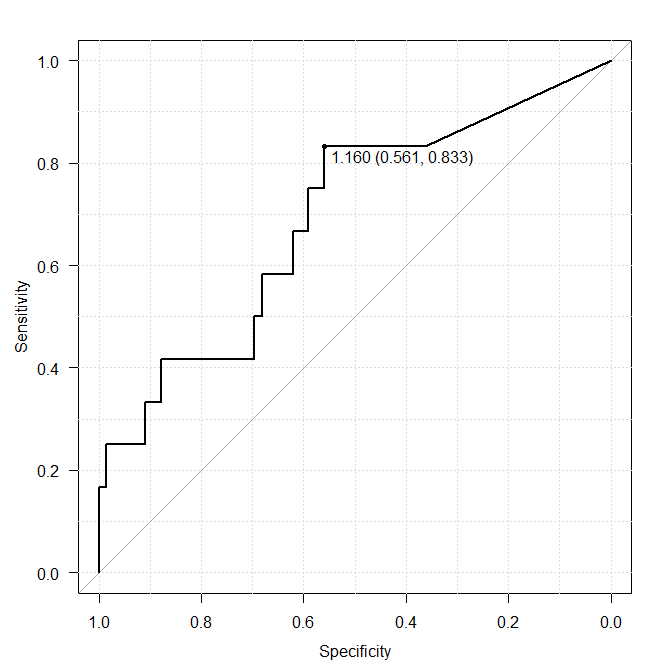

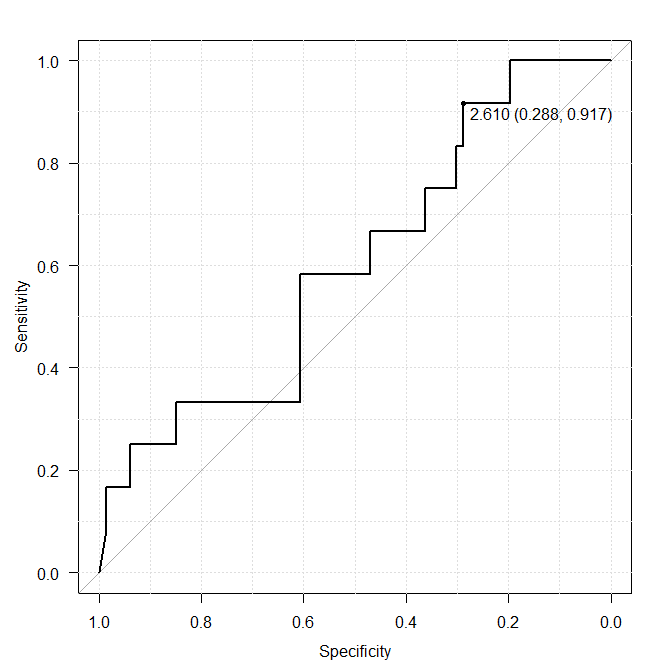

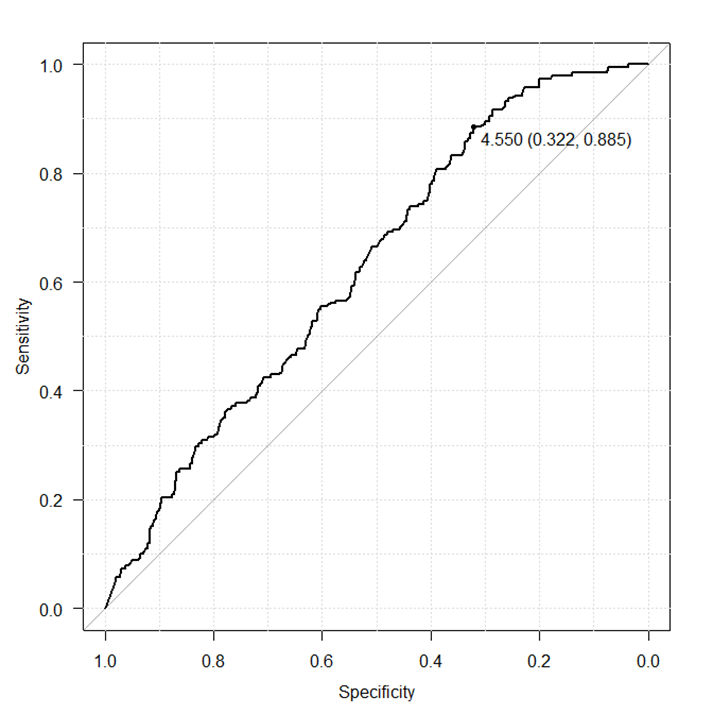

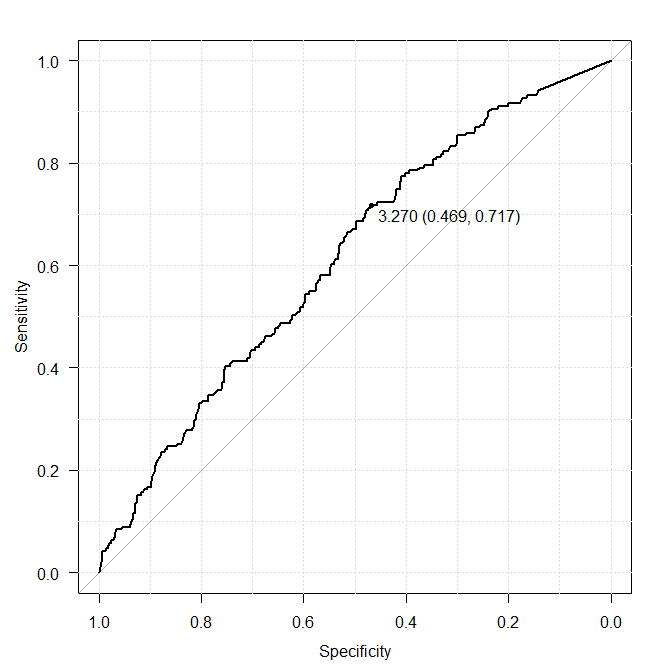


**EW-sIgE**

**OVMsIgE**

**All**

**Complete elimination**

**Partial elimination**
